# Revision on Psychometric Properties of the Temperament and Character Inventory in a Clinical Sample

**DOI:** 10.3389/fpsyg.2018.01951

**Published:** 2018-10-12

**Authors:** Silvia Dell’Orco, Raffaele Sperandeo, Enrico Moretto, Nelson Mauro Maldonato

**Affiliations:** ^1^Department of Humanistic Studies, University of Naples Federico II, Naples, Italy; ^2^School of Integrated Gestalt Psychotherapy, Torre Annunziata, Italy; ^3^Department of Neuroscience and Reproductive and Odontostomatological Sciences, University of Naples Federico II, Naples, Italy

**Keywords:** Temperament and Character Inventory, personality disorders, vulnerability, anxiety, prefrontal cortex

## Abstract

Cloninger’s Temperament and Character Inventory (TCI) although elaborated on the general population, is frequently used in clinical samples. The study evaluates the psychometric characteristics of TCI in clinical populations with the aim of creating a reduced version of the test suitable for these subjects. This research was conducted on two groups of mental health outpatients. In the first study, 44 items, correlated with the psychiatric disorders, was selected. These items, divided in four dimensions utilizing both statistic and psychopathological criteria, show good internal consistency and external validity and constitute a Reduced Version (TR-TCI) of the test. In the second study, the predictive validity of the TR-TCI was evaluated through the ROC curves and a logistic regression model. The results show a good predictive validity of TR-TCI, that allows us to use this instrument in order to identify the personality structures that make people sensitive to psychiatric pathologies.

## Introduction

The use of interviews or self-report questionnaires for the diagnosis of mental and personality disorders has entered daily clinical practice. Scientific progress in psychopathology but also therapeutic procedures are increasingly dependent on this diagnostic practice. The transformation of the symptoms of psychiatric disorders into items of an assessment scale requires a methodology that is still not well defined. Traditional psychometric methods, based on Error Theory, (Kline, 2013) that have been used for the definition of personality assessment tools are not suitable for measuring psychiatric symptoms; in fact, it is not clear what characteristic or property of a symptom is described by an item or how it measures intensity and severity of a symptom (Berrios and Marková, 2015). In fact, unlike the items that describe behaviors and personality characteristics that can be constructed with dichotomous answers, the items describing the symptoms need vector responses to describe the intensity of the pathological manifestation. The construction of valid vector responses poses problems of stability and validity that have not yet been solved (Berrios and Marková, 2015).

It is evident, therefore, that the psychometric instruments used in the clinical field must reconcile the requirements of validity and reliability with the mutability and internal structure of the psychopathological symptoms. In fact, the diagnostic needs of organic medicine differ profoundly from those of psychiatry. The diagnostic question that originates from the practice of body medicine, and from the semibiotic connected to it, is the following: from which pathology is the person before us affected and how serious are its clinical manifestations? The deterministic value of this question, when it was transferred to psychiatry, has created significant problems in the nosographic definition. It has generated the illusion of describing a psychiatric disorder as a summation of “diagnostic criteria” by creating artificial nosographic constructs called “categorical diagnoses.” This is indeed the structure of the current psychiatric nosographic manuals and specifically of the DSM IV-TR (American Psychiatric Association, 1996) which classifies psychiatric disorders in:

Axis I disorders that include mood disorders, anxiety disorders and psychosis and other psychiatric disorders
Axis II disorders that include, among others, personality disorders

The diagnostic frameworks provided by categorical classifications, however, solve the problem of communication between clinicians but do not meet the needs of the psychiatric clinic and offer scientific research objects that are fictitiously closer to conceptual categories than to natural pathological entities.

To remedy the difficulties presented, it is useful in clinical and research contexts, alongside psychiatric scales, also to assess the personality with the aim of providing an integrated description of the patient in question. In this way, we can answer the question: how is the person before us and which individual characteristics determine the state of discomfort? At present this question is the most suitable for clinical practice because it allows for the definition of the pathology from which the patient is affected, in relation to his personal specificities (Berrios, 2013).

An important widespread tool in the clinical field that allows a “dimensional reading” of the person was developed in the early nineties by C. Robert Cloninger, and his group, and has given rise to a vastup body of scientific literature inspiring numerous researchers. It is a psychobiological dimensional model of personality, structured on two levels, defined as temperament and character. It places the normal and pathological aspects of personality on the same continuum, the normal and pathological aspects of the personality and characterizing as pathological the extreme expressions of the dimensions of temperament and character (Cloninger, 1992, 1994; Cloninger et al., 1993; Gutierrez-Zotes et al., 2004). Temperament is considered as the emotional heart of personality, refers to individual differences in automatic responses to environmental stimuli. According to Cloninger, these are inheritable components, observable early in childhood and not influenced by socio-cultural learning and include four independent dimensions: (1) Novelty Seeking (NS), which represents individual differences in behavioral activation in response to novelty; (2) Harm Avoidance (HA), which represents individual differences in behavioral inhibition in response to danger or punishment signals; (3) Reward Dependence (RD), which represents individual differences in the search for socially rewarded behavior; (4) Persistence (P), which represents individual differences in the ability to persevere in their goals despite fatigue and frustration. Character is described as an explicit model of consciousness (Maldonato et al., 2018a) and assumed conceptual expectations in relation to personal goals, life in the world and others. It is defined in terms of individual differences in self-experience that changes throughout the lifespan in response to socio-cultural influences. It includes three dimensions:

(1) Self-Directedness (SD), based on a representation of self as an individual from which derive feelings of personal integrity, honor, self-esteem, personal effectiveness, leadership and hope;
(2) Cooperativeness (C), based on a representation of self whether as an integral part society or humanity, from which derive feelings of community, compassion, charity and conscience;
(3) Self-Transcendence (ST), based on a representation of itself as an integral part of the universe from which derive feelings of patience, mystical participation and religious faith (Maffei, 2008; Maldonato et al., 2018b).

Cloninger believes that ethological studies suggest that personality development evolves based on pre-semantic experiences related to temperament. The semantic reorganization of experience and the concept of self are the results of learning new adaptive answers.

In insight-based learning, our unconscious, automatic responses, initially determined by the genetic temperamental factors, are, subsequently, modified by the continuous reorganization of the representation of oneself and of one’s own identity and by the changes in meaning that derive from it. The development of the personality is, from this perspective, a process in which the hereditary temperamental factors initially motivate conscious learning and structure the concept of self, which, in turn, modulates the meaning and importance of the stimuli to which the individual answers. Temperament and character development influence each other and motivate behavior (Manna, 2012). As argued by Cloninger himself, the evolution of the human brain involved the development of five major adaptive systems that allow conscious regulation of the sexual, material, emotional (Maldonato and Dell’Orco, 2015), intellectual, and spiritual aspects of life experience (Cloninger, 2009). These systems appear in a close interaction with environmental events in a continuous process of circular causation. Thus the distinction that categorical psychiatric models operate between different systems (such as the I and II axes of the DSM) does not appear to be consistent with the non-linear mode of brain functioning (Cloninger, 2004; Sperandeo et al., 2018b). The psychobiological model of Cloninger which is described by a specific self-report diagnostic reagent (Temperament and Character Inventory, TCI) has been widely analyzed in relation to numerous psychiatric pathologies. In particular, correlations between mood disorders (Miettunen and Raevuori, 2012; Zaninotto et al., 2016) and high scores at the temperamental dimension defined Harm Avoidance were found (Young et al., 1995; Osher et al., 1996; Engström et al., 2004; Nowakowska et al., 2005; Zaninotto et al., 2015). A recent meta-analysis concerning the temperamental characteristics of subjects with psychiatric disorders (Miettunen and Raevuori, 2012) showed that subjects with both unipolar and bipolar mood disorders had high scores at HA size and that subjects suffering from Major Depressive Disorder (Esposito et al., 2016; Sperandeo et al., 2018a) score low in the temperamental dimensions defined as Novelty Seeking and Reward Dependence.

However, meta-analyzes present numerous methodological and statistical force problems. In particular, as far as we are concerned, they cannot discriminate the presence of certain confounding factors such as socio-demographic variables (Wasek and Endicott, 1983; Cloninger, 1992; Cloninger et al., 1993; Peselow et al., 1995; Gutierrez-Zotes et al., 2004; Hansenne et al., 2005; Mikołajczyk et al., 2008; Chen et al., 2013) and the number of psychopathological episodes in the arc of life (Hirschfeld et al., 1989; Zaninotto et al., 2015; Paolini et al., 2016). Finally, meta-analyzes should also include follow-up studies as these can help to disentangle the “state-trait” effect to determine if a specific character vulnerability model exists for the development of affective disorders (Farmer et al., 2003).

According to Cloninger it is possible to predict the presence of a personality disorder in subjects who obtain scores below 33 percentage points to the character dimensions defined as Self-Directedness and Cooperativeness. This score was obtained from previous studies in clinical samples in which low scores were reported on the size of SD and C in subjects with Personality disorder (Cloninger, 1992, 1994). This evidence was confirmed in a study by Paolini et al. (2016), who used TCI in a population of outpatients and found one correlation between the diagnosis of Personality Disorders and scores below 27 on the Self-Directedness scale and below 29 on the Cooperativeness scale (corresponding to 33% of the maximum value). TCI was also used to study the correlations between neurobiological activity and vulnerability to psychiatric disorders and used to investigate the neurobiological foundations of personality correlating it with the studies of functional neuroimaging, structural neuroimaging and genetics (Pujol et al., 2002; Garcia-Romeu, 2010; Garcia et al., 2013). Studies showed that higher HA (Harm Avoidance) scores were associated with a smaller regional gray matter volume in the right hippocampus. In contrast, a female-specific correlation was found between personality traits related to anxiety and the lower regional volume of the brain in the left anterior prefrontal cortex. The results may have important implications because the susceptibility to stress-related disorders such as anxiety disorders and depression show individual differences related to sex (Yamasue et al., 2007). Despite the clinical relevance and its good predictive validity of mood disorders and personality disorders, the TCI shows controversial psychometric properties. TCI reliability studies report consistent reliability ratios in many situations. In general, the reliability coefficients (Cronbach’s internal Alpha consistency and test–retest reliability) for TCI scales are adequate in clinical and non-clinical samples, although the RD and P scales show a low internal consistency in non-clinical samples (Maffei, 2008).

Factor analyzes show controversial results about the latent structure of TCI. When we proceed with the joint factorial analysis of the Temperament and Character scales, some dimensions do not emerge as distinct factors and the Temperament and Character dimensions overlap each other, suggesting that the conceptual distinction between the two scales is not empirically supported. Despite widespread use and the clinical relevance of the questionnaire, in total, these findings suggest the need for more revisions of the TCI. In 1999, Cloninger developed the TCI-R (Maffei, 2008). Analyzing the most recent studies (Wong and Cloninger, 2010), it emerges that the TCI-R has an internal consistency and an adequate test–retest reliability. From the point of view of the dimensions of the personality, the data support the seven-factor model although two aspects should be emphasized: (1) no joint factorial analysis of temperament and character was carried out; (2) a mixed sample (clinical and non-clinical) was used for validation. The psychometric properties of TCI-R in its Italian version were evaluated using two independent samples, a clinical sample and a non-clinical sample. Furthermore, a moderate convergence between the TCI and TCI-R scales was observed in the clinical sample. Once these correlations were corrected for mitigation due to measurement error, convergence grew significantly for all scales (Maffei, 2008). Cloninger et al. (1993) reported the internal coherence coefficients for the main scales and for the TCI facets based on a sample of 300 volunteer buyers at a shopping center. Although there was some improvement in the internal coherence coefficients for the four main temperament scales (range of α of C: 0.65 to 0.87), the consistencies internal of facet scale tended to be modest (range: 0.54 to 0.76). The internal coherence coefficients for the three various main character sizes were adequate (range: 0.84 to 0.89), but were relatively modest for the associated facets (range: 0.47 to 0.86).

Svrakic et al. (1993) reported similar coefficients of internal coherence in samples of psychiatric patients. The results of TCI factorial analysis are noteworthy, as they have revealed a mixture of Temperamental and Characteristic factors and facets that do not always support the conceptual distinctions of Cloninger.

## Objectives Of The Study

The goal is to create a small, agile diagnostic tool from TCI using only the statistically relevant items using outpatient clinical samples, organizing the items into homogeneous groups from a clinical point of view and consistent in terms of the theoretical model. A test of this type can be used as a screening tool to identify clients with Axis I disorders.

## Materials and Methods

### Temperament and Character Inventory (TCI)

To evaluate the Cloninger model, the tool that the author has developed was used: the TCI. The TCI is a self-assessment questionnaire (self-report) which, in its complete version, consists of 240 dichotomous answer items (true/false). Of these, 116 explore the four temperamental dimensions (NS, HA, RD, and P) and 119 evaluate the three dimensions of the character (SD, C, and ST). The sum of the items marked as “true” provides the raw score of the seven scales. The raw scores are transformed into standardized *T*-scores which, shown on a chart, provide a personality profile of the subject (Manna, 2012). This tool includes questions about tastes, interests, emotions, answers, goals, and values (Wong and Cloninger, 2010). The results of the TCI can be evaluated as raw score, *T*-score and percentage score, and a conversion table is provided between these three measures based on the score obtained in a standardization on a sample of 300 adults, called community champion; Cloninger states that it is representative of the general population and supports the reliability and structure of the TCI dimensions (Cloninger et al., 1993).

### Mini International Neuropsychiatric Interview (MINI)

The MINI ^∗^ is a semi-structured diagnostic evaluation scale developed jointly by the Sheelan (United States) and Lecrubier (France) research teams. It is short, simple, clear and easy to administer; is highly sensitive and able to identify the highest possible percentage of subjects with a given disorder, is selective and able to exclude subjects without disorders, compatible with the main international diagnostic classification systems, ICD-10 and DSM-IV-TR; finally, it is able to capture the most important sub-syndromic variants. To avoid an excess of false negatives, the authors designed the scale o that each of the disorders investigated was slightly hyperinclusive^∗^.

Each disorder investigated corresponds to an autonomous form; most of the modules include one or two preliminary screening questions, the negativity of which allows the complete exclusion of the symptomatology related to that disorder and allows the subject to go directly on to the next module. When the patient responds positively to the screening question (s), they instead move on to questions associated with the detection of symptoms, which should be completed by questions about the disability associated with those symptoms, on the possible concomitance with somatic pathologies and/or use of substances, on any recent losses. The modules include 14 Axis I disorders, an Axis II disorder, antisocial personality disorder (including its stability over time, the consistency shown in various personality disorders and its impact on the clinic and prognosis) and a form relating to suicide risk (Rossi et al., 1998; Sheehan et al., 1998).

### Samples

The subjects belonging to the “experimental sample” have a mean age of 34.7 years (*SD* = 11.48).

Of the 525 subjects belonging to this group: 57 do not present any disorders; 266 have a diagnosis of major depressive disorder; 27 of dysthymia; 193 of panic disorder; 249 of generalized anxiety disorder; 68 of obsessive-compulsive disorder; 26 of bipolar disorder; 17 of social phobia; 16 of alcohol abuse disorder; 22 had diagnoses of substance abuse disorder; 27 have psychotic disorder; 13 of eating disorder; finally, 71 subjects fall within the exclusion criteria. 233 are males and 292 are females; 37 completed elementary school, 104 middle school, 284 had achieved high school diploma, 99 achieved degree at university, and only 1 participant has no qualification. In relation to marital status, 33 are separated or divorced, 12 are widowed, 284 are single or unmarried, 196 are married, 362 subjects are employed in work or study, 155 are unemployed, and 8 are pensioners, 71 subjects have been eliminated because they fall within the exclusion criteria 125 subjects have a single diagnosis of Axis I, 155 have two diagnoses, 100 have three diagnoses, 17 have four diagnoses.

The “verification group” consists of 324 subjects. Of these: 83 do not present any disorders; 109 have diagnoses of major depressive disorder; 17 of dysthymia; 77 of panic disorder; 95 are of generalized anxiety disorder; 27 of obsessive-compulsive disorder; 12 of bipolar disorder; 8 of social phobia; 7 of alcohol abuse disorder; 10 of substance abuse disorder; 13 have psychotic disorder; 6 have eating disorder. 83 subjects have only one Axis I diagnosis, 99 have two diagnoses, 49 have three diagnoses, 10 have four diagnoses; 143 are males and 181 are females; 22 completed elementary schools, 64 middle schools, 175 high school, 61 graduates; 2 do not have any qualifications. In relation to marital status, 20 are separated or divorced, 9 widows, 177 are single or unmarried, 118 are married. 225 subjects are employed in work or study, 94 unemployed and 5 are pensioners.

Socio-demographic and clinical status of the samples are described in **Tables 1**, **2**.

**Table 1:** Socio-demographic status of the samples.

	Experimental sample	Verification sample
**No. of subjects of the samples**	525	324
Mean age	34.7 (±11.48)	33.96 (±11.22)
Socio-demographic variables	No. of subjects	No. of subjects
Male	233	143
Female	292	181
Employed -	362	225
Unemployed	155	94
pensioners	8	5
Singles	284	177
Married	196	118
Separated or divorced	33	20
Widowed	12	9
Elementary school diploma	37	22
Middle school diploma	104	64
High school diploma	284	175
Degreed at university	99	61
No qualifications	1	2

**Table 2:** Clinical status of the samples.

	Experimental sample	Verification sample
Psychiatric disorders	No. of subjects	No. of subjects
Major depression	266	109
Dysthymia	27	17
Panic disorder	193	77
Generalized anxiety disorder	249	95
Obsessive-compulsive disorder	68	27
Bipolar disorder	26	12
Social phobia	17	8
Substance abuse disorder	38	17
Psychotic disorder	27	13
Eating disorder	13	6
No psychiatric disorder	57	83
Excluded from the study	71	0
Subjects with a single psychiatric disorder	125	83
Subjects with two psychiatric disorder	155	99
Subjects with three psychiatric disorder	100	49
Subjects with four psychiatric disorder	17	10

### Methodology

To perform the study it was preferred to use the TCI in its first version which is superimposable, in clinical samples, to the TCI-R latest version of the instrument (Maffei, 2008).

The choice of a test with dichotomous response was determined by two reasons. From a clinical point of view, the scalar response is less pleasing to patients than dichotomous because of the greater difficulty they find in selecting the right score on the scale of values. Under a psychometric profile, items with dichotomous response have a lower risk of occasional error because they are less influenced by contingent events, can offer a better reliability test–retest and consequently, a superior validity and reliability (Kline, 2013).

The choice to use the individual TCI items rather than the seven factors of the model was determined by the need to reformulate the factorial structure of the tool for its use in psychiatric patient populations. Diagnostic reagents produced by factor analysis lose much of their validity when used in samples dissimilar to those in which they were generated. This depends on the fact that the statistical test does not guarantee a correspondence with the theoretical model of reference, since the weights of the components of the factors are generated by mathematical procedures, obviously, not connected to the psychological theory. In fact, the TCI has been constructed in the general population and its factorial structure does not make it suitable for use in the psychiatric patient population.

Finally, to further support the choice of using the individual items of the test, it is useful to take into account the fact that they have an explicit external validity, superior to that of the factor dimensions. In fact, the items of a personality questionnaire, that require the subject to say whether or not they adhere to a certain behavior or thought, trigger the activation of the same neuronal network that is involved in the actual execution of the described behavior. The activation of this network gives rise to the formulation of a concept characterized by a certain level of abstraction but embodied in the ideomotor processes of the subject in order with “enacted theory” of mind (Caruana and Borghi, 2016).

In our opinion, the individual items of a questionnaire can be effectively used to explore the mental contents typical of a personality style, because they have criteria of concreteness and ecologicality that are lost when the items are organized into factors (Maldonato et al., 2016; Sperandeo et al., 2017).

For this purpose, of the 240 items of the test, only items proven in the sample under examination to be related, negatively or positively, with the presence of psychiatric pathologies of Axis I were selected. The items selected on the basis of the correlation test were finally further reduced based on their psychopathological consistency. The criterion for evaluating psychopathological coherence was based on the mixed personality model proposed by the DSM 5 in appendix 3. This model organizes the functioning of the personality on two levels: the self, understood as integration between identity and self-directionality, and interpersonal relationships, intended as integrations between empathy and the capacity for intimacy (Manna, 2012).

The items selected with a correlation test have been merged and further skimmed according to the criteria described by American Psychiatric Association (2013).

The study was conducted on two different samples of subjects who have been consecutively registered in the last 5 years in two different services for the outpatient treatment of mental disorders located in the province of Naples. The first sample called “experimental sample,” consisting of 397 subjects with psychiatric disorders of Axis I and 57 healthy subjects, was used to structure the TCI Reduced Test (TR-TCI), to evaluate the internal consistency and the incremental validity in the contexts clinical trials compared to the TCI first version.

The second sample called “verification sample,” consisting of 241 subjects affected by psychiatric disorders of Axis I is 83 healthy subjects, was used to verify the predictive validity TR-TCI.

In a first phase, in the “experimental sample” all the TCI items were compared with the presence of Axis I pathologies. The 54 items that were significantly correlated, positively or negatively, to the presence of psychiatric disorders were used to create the new ones homogeneous dimensions of the TR-TCI personality. All the items that were descriptive of temperament in the TCI were divided into two groups: the first composed of those positively correlated with the presence of psychiatric pathologies, the second composed of those negatively correlated with the presence of psychiatric pathologies. The descriptive items of the character, all positively correlated with the presence of psychiatric pathologies, were divided into two groups on the basis of their coherence with the psychopathological theory of reference. Finally, in all four groups, non-coherent psychopathological items were considered as artifacts of the correlation test and were eliminated. Through this procedure, 44 items were identified divided into four dimensions of the personality: called: Optimism (O), Closeness to Experience (CE), Passivity (P), and Superiority Fantasy (FS). For each of the four dimensions, consisting of 6, 13, 14, and 11 items, respectively, the value of Cronbach’s alpha was measured. Each item was assigned the value 1 for the “true” answer and the value 0 for the “false” answer and the sum of the scores for each item and a maximum score was obtained for each of the dimensions. Finally, the variance of the total score of the four dimensions was analyzed to evaluate its external validity and the incremental validity in clinical samples of TR-TCI compared to TCI. In a second phase, the four dimensions of the TR-TCI created in the experimental sample were evaluated with respect to the predictive validity in the “test sample.”

### Sampling Procedures

The subjects of the two samples used in the study were sequentially assessed by two outpatient services for the treatment of psychiatric pathologies. Upon entry into treatment, the subjects were determined to have an Axis I diagnosis according to the DSM IV-TR criteria using the MINI. Furthermore, a diagnosis of Axis II disorders was performed using the SCID II Semi-Structured Diagnostic Interview that provides diagnosis of Personality Disorders According to the DSM IV TR criteria. Finally, the subjects completed the TCI for the evaluation of the personality according to the Cloninger model.

Subjects suffering from Personality Disorders, subjects with cognitive deficits and histories of cranial traumas were excluded from the study in line with the diagnostic criteria of psychiatric disorders of the DSM 5.

All subjects were informed and accepted that the data collected for the clinical evaluations would be used in scientific studies according to the legislation regarding the respect of anonymity and confidentiality of sensitive information and the assurance that use of the data would not modify the treatment prescribed.

### Statistics

The “Internal Consistency” of the four new personality dimensions identified by the TR-TCI was evaluated through Cronbach’s alpha measurement. “External validity” was evaluated by analyzing, through the ANOVA, the means of total scores in subgroups of patients with and without mental disorders. The results were compared with those obtained from the factor size of the TCI. The comparison between the two tests (TCI and TR-TCI) was repeated in the “test sample.”

Finally, in the “verification sample,” the “predictive validity” of the TR-TCI was evaluated through the production of ROC curves and the creation of a “logistic regression” model in which the presence or absence of psychiatric pathologies represent the dependent variable. In the creation of the regression model, the scores of the “Optimism” dimension were inverted.

## Results

Table 3 shows the values of Spearman correlation coefficients for the 54 TCI items negatively or positively related to the presence of psychiatric disorders. The rho values are low but the correlations are statistically significant and this makes them relevant to the use of the diagnostic tool in clinical samples. The low value of the correlation coefficient is consistent with the psychopathological theory that predicts that the personality structure has a reduced weight in the development of Axis I disorders (Table 3).

**Table 3:** Items related positively and negatively to psychiatric disorders.

**26 item descriptors of temperament significantly correlated with the presence of psychiatric disorders Spearman’s Rho correlation**														

Item number in the TCI	114	144	238	35	174	91	165	183	2	20	65	149	164	
Presence of Axis I disorders	**0.09**	**0.09**	**0.11**	**0.10**	**-0.10**	**0.09**	**-0.08**	**0.08**	**-0.09**	**0.12**	**-0.12**	**0.14**	**-0.16**	**Rho**
Item number in the TCI	225	12	129	217	80	142	22	43	63	92	113	147	205	
Presence of Axis I disorders	**0.10**	**0.15**	**0.18**	**0.17**	**-0.11**	**-0.12**	**0.10**	**0.09**	**0.13**	**0.16**	**0.17**	**-0.09**	**0.09**	**Rho**

**28 descriptive character items significantly correlated with the presence of psychiatric disorders Spearman’s Rho correlation**														

Item number in the TCI	4	86	169	9	126	40	106	197	32	60	74	94	107	150	
Presence of Axis I disorders	**0.08**	**0.11**	**0.08**	**0.12**	**0.12**	**0.14**	**0.14**	**0.13**	**0.13**	**0.16**	**0.13**	**-0.10**	**0.11**	**0.11**	**Rho**
Item number in the TCI	179	229	39	104	162	184	221	16	48	122	234	146	75	176	
Presence of Axis I disorders	**0.09**	**0.15**	**0.15**	**0.14**	**0.15**	**0.09**	**0.08**	**0.12**	**0.09**	**0.12**	**0.09**	**0.09**	**0.12**	**0.08**	**Rho**

*Bold are the significant values of factor analysis*.

**Table 4** shows the structure of the four dimensions of the TR-TCI. It contains the items that make up the new dimensions of the personality that have a significant internal consistency measured by the Cronbach test, with alpha values between 0.66 and 0.87. The low value (0.66) of Cronbach’s alpha of the “O” dimension is accounted for by the low number items (6) that make up this dimension. The four dimensions are partially correlated with correlation coefficients between 0.17 and 0.63 with a correlation average of 0.43 and a reliability coefficient with respect to the 0.66 error and a Cronbach’s alpha of 0.75.

**Table 4:** Description of the four dimensions of the TCI Reduced Test (TR-TCI).

TEMPERAMENTAL DIMENSIONS	
**OPTIMISM (OTT)** *Cronbach’s alpha = 0.66*	
**No. of item**	Those who respond positively to these ITEMS are optimistic about future events and situations that worry most people, have a strong belief in their abilities and a tendency to make rapid decisions
1 (2)	I trust that everything will be all right, even in situations that worry most people
2 (65)	Regardless of the problems I face, I think everything will be fine
3 (80)	I would feel relaxed meeting strangers, even unfriendly ones
4 (142)	I feel confident and confident in all social situations
5 (147)	I get tired less than most people
6 (164)	I do not worry that something bad will happen in the future
**CLOSENESS TO EXPERIENCE (CE)** *Cronbach’s alpha = 0.80*	
**No. of item**	Subjects who respond positively to these ITEMS tend to reject new stimuli and have lived anxious and pessimistic anticipations in relation to unusual activities. They do not actively seek gratification, and prefer monotonous and habitual lifestyles.
1 (12)	I feel tense and worried in unusual situations, even when others think that there is nothing to worry about
2 (20)	I often interrupt my activities because I worry that they could go wrong
3 (22)	I get tired more quickly than others
4 (35)	I find it difficult to keep the same interests for long, because my focus shifts to something else
5 (43)	I recover more slowly than others from small ailments or stress
6 (63)	I need to rest often because I get tired easily
7 (92)	I need rest and support to recover from small illness or stress
8 (113)	It’s hard to adapt to changes because it makes me tense, tired or worried
9 (129)	I feel tense and worried, even in situations where others think there are no dangers
10 (144)	I hate to change my way of doing things, even when there is a better one
11 (149)	I often stop doing something because I start to worry about making mistakes, despite the reassurances
12 (217)	I feel tense and worried if I have to do something new
13 (225)	Things go wrong if I’m not very careful
**CHARACTERIAL DIMENSIONS**	
**TENDENCE TO LIABILITIES (TL) –** *Cronbach’s alpha = 0.84*	
**No. of item**	Those who respond positively to these items perceive themselves as incapable of modifying the world around them and are convinced that their existence has little value for others
1 (4)	I feel a victim of the circumstances
2 (9)	I feel that my life has little meaning
3 (39)	I have bad habits that I would like to change
4 (40)	I wait for someone else to provide a solution to my problems
5 (86)	Others control me too much
6 (104)	I do not appreciate all the faults I have
7 (106)	I cannot solve my problems because I do not know what to do
8 (126)	I do not have a clear idea of the ultimate goal of my life
9 (162)	My habits make it difficult to complete important goals
10 (169)	My actions are determined by influences from my control
11 (176)	My brain does not work well
12 (184)	I need to apply a lot to get good habits
13 (197)	People have more resources than I have
14 (221)	My will is too weak to win strong temptations even though I know I will suffer as a result
**SUPERIORITY FANTASY (FS)** *– Cronbach’s alpha = 0.76*	
**No. of item**	Subjects who respond positively to these items want to be superior to others and tend not to accept the diversity of others and the passage of time
1 (16)	I do not like people with different ideas than mine
2 (32)	I’d like to be smarter than anyone
3 (48)	I have no patience with those who do not accept my points of view
4 (60)	I would like to be stronger than anyone
5 (74)	I would like to be young forever
6 (107)	I would like to be able to stop the flow of time
7 (122)	It is difficult to bear people different from me
8 (146)	I like to imagine my enemies suffer
9 (150)	I would like to be more powerful than anyone
10 (229)	I’d like to look better than anyone
11 (234)	Those who are dealing with me must learn to do things in my way
The numbers of the TCI items are shown in brackets	**Internal reliability among dimensions (FS, TL, OTT, CE)** Cronbach’s alpha = 0.75

The 44 items were selected through the procedure described in the section “Methodology.” The 19 items derived from the temperament of the TCI were divided into two groups: the first, labeled as “Optimism” is composed of 6 items for which the “true” choice was negatively correlated to the presence of disease; the second labeled “Closeness to Experience” consists of 13 items for which the “true” choice was positively correlated with the presence of the disease.

The “O” dimension describes subjects that are markedly optimistic with respect to the future, with great confidence in their own resources and determined to action. The “CE” dimension describes subjects that avoid new stimuli have a marked anxiety, a tendency toward pessimistic anticipation and habitual behaviors and reflexivity, a poor search for rewards and refusal for unusual activities.

The 25 items derived from the character domains of the TCI, all characterized by positive correlation with the presence of psychiatric disorders, were divided into two dimensions on the basis of psychopathological evaluations. The first dimension called “Tendence to Liabilities” describes subjects who represent themselves as incapable of changing their own reality, which is basically not very autonomous and with little sense of the value of their existence. The second dimension called “Superiority Fantasy” describes subjects who fantasize, narcissistically, to be more intelligent, attractive and stronger than anyone else and fear the passage of time and the weakening of the body (**Table 4**). The analysis of the variance (ANOVA) carried out in the experimental sample for the seven dimensions of the TCI and the four dimensions of the TR-TCI (**Table 5**). The scores averaged from 57 subjects without psychiatric disorders were compared with the averages obtained from 397 subjects with at least one Axis I disorder. The four dimensions of the TR present significantly different averages in the two subgroups. The TCI scores significantly different only in the dimensions: Harm Avoidance and Self-Directiveness (**Table 5**).

**Table 5:** Dimensions of the TCI and the TR-TCI comparison in the experimental sample.

TCI DIMENSIONS	PSYCHIATRIC DISORDERS	*M*	*SD*	*F*	*p*	η
Novelty Seeking	Present	50.07	14.18	0.27	n.s.	0.00
	absent	50.84	15.29			
Harm Avoidance	Present	57.42	14.44	34.28	<0.01	0.24
	Absent	48.95	13.58			
Reward Dependence	Present	62.65	15.61	0.306	n.s.	0.00
	absent	61.79	14.72			
Persistence	Present	55.29	23.31	0.184	n.s.	0.00
	Absent	54.30	20.9			
**Self-Directedness**	**Present**	**53.93**	**13.57**	**11.16**	**<0.01**	**0.14**
	**Absent**	**58.47**	**12.63**			
Cooperativeness	Present	71.25	14.74	2.11	n.s.	0.00
	Absent	73.44	14.91			
Self-Transcendence	Present	37.41	17.2	0.747	n.s.	0.00
	Absent	39.01	20.9			

**TR-TCI DIMENSIONS**	**PSYCHIATRIC DISORDERS**	***M***	***SD***	***F***	***p***	**η**

Closeness to Experience	Present	7.27	3.26	45.45	<0.01	0.28
	Absent	5.03	3.23			
Optimism	Present	1.67	1.58	26.34	<0.01	0.22
	Absent	2.52	1.74			
Tendence to Liabilities	Present	6.59	3.76	15.98	<0.01	0.17
	Absent	5.05	3.76			
Superiority Fantasy	Present	4.33	2.6	10.55	<0.01	0.14
	Absent	3.46	2.78			
**Average η (Student *t*-test)**	TCI	0.054	0.09	*T*	*p*	
	TR	0.2	0.06	**23.95**	**<0.05**	

*Bold are the significant values of factor analysis*.

In the second phase of the study, the reduced test was evaluated in a sample of 324 subjects labeled as an “evaluation sample,” with the aim of documenting the ability of this tool to predict the presence of psychiatric Axis pathologies.

**Table 6** shows the analysis of variance (ANOVA), carried out in the test sample, for the four dimensions of the TR-TCI and the two dimensions of the TCI results correlated to the pathology in the experimental sample. The averages of the four dimensions of the TR-TCI and the two dimensions of the TCI were significantly different in the two subgroups, as already noted in the experimental sample. The values ?of Eta also document, in this sample, a greater Effect Size for the Reduced Test size.

**Table 6:** Dimensions of the TCI and TR-TCI comparison in the verification sample.

DIMENSIONS	PSYCHIATRIC DISORDERS	*M*	*SD*	*F*	*p*	η
Harm Avoidance	Present	56.10	13.96	30.22	<0.01	0.17
	Absent	47.01	13.26			
Self-Directedness	Present	56.25	12.98	8.81	<0.01	0.16
	Absent	60.82	12.38			
Closeness to Experience	Present	6.80	3.24	37.23	<0.01	0.23
	Absent	4.48	3.05			
Optimism	Present	1.79	1.58	26.41	<0.01	0.16
	Absent	2.79	1.76			
Tendence to Liabilities	Present	5.92	3.55	18.05	<0.01	0.27
	Absent	4.15	3.30			
Superiority Fantasy	Present	3.75	2.43	8.82	<0.01	0.32
	Absent	2.88	2.5			

Specifically, optimistic temperament is more often associated with subjects without pathologies, where Closeness to the experience is more often associated with subjects presenting psychiatric disorders. Likewise, the character dimensions “passivity” and “Superiority Fantasy” are expressed in a more intense way, on average, in the subgroup of subjects with at least one psychiatric disorder (**Table 6**).

**Table 7** shows the values of the ROC curves with which the four dimensions of the TR-TCI were evaluated in relation to their sensitivity to the presence of psychiatric pathologies.

**Table 7:** Sensitivity and specificity of the reduced test size vs. the presence of at least one Axis I disorder.

Curves ROC				
Variables	Area under the curve	Sign. asymptotic	95% asymptotic confidence interval	

			Lower limit	Upper limit
Closeness to Experience	0.7	<0.01	0.64	0.76
Optimism	0.66	<0.01	0.60	0.73
Tendence to Liabilities	0.65	<0.01	0.58	0.71
Superiority Fantasy	0.62	<0.01	0.55	0.69

The values of the area under the curve document a high sensitivity of the “CE” temperamental dimension, an average sensitivity of the dimensions: “O,” “P,” and “FS” (**Table 7**).

**Table 8** shows the logistic model of prediction of the presence of pathology of Axis I. The increase of a point to the dimension “CE,” or the reduction of a point to the dimension “O” allow to foresee an increase of the risk of psychiatric pathology, respectively, 22% and 17%. Finally, as shown by the values of R^2^, a high percentage of the variance is explained by the model that also allows the scale to obtain 74.8% of global correct forecasts (**Table 8**).

**Table 8:** Logistic regression dependent variable: presence of at least one Axis I disturbance – explanatory variables.

Explanatory variables	*B*	*SE*	*p*	Exp (*B*)		*R*^2^ Cox and Snell	*R*^2^ Nagelkerke
Optimism	0.17	0.02	<0.01	1.19		0.27	0.36
Closeness to Experience	0.22	0.06	<0.01	0.25			

Percentage of correctness of the classifications							

Presence of at least one Axis I diagnosis				Absent	34.0%		
				Present	91.3%		
Global percentage				74.8%			

## Discussion

Of the 240 items of the TCI only 44 were used for the construction of four personality dimensions closely related to the presence of psychiatric disorders of Axis I. Nineteen of these items belong to the temperamental dimensions and 25 to the character dimensions of the TCI. The dimensions of the TR-TCI were composed by respecting the character and temperament derivation of the items. Specifically, the items derived from the size of the temperament were divided into two groups that showed they had a good “internal consistency.” The first, called “closeness to experience” is composed of items for which the “true” answer is positively correlated with the presence of psychiatric pathologies of Axis I and seems to identify this factor in the personality structure a weak point that makes people not adaptable to environmental pressures typical of our culture. The second dimension defined “Optimism” is composed of a few items for which the “true” answer is negatively correlated with the presence of psychiatric pathologies and seems to identify a trait of the adaptive personality that makes people resilient to stress.

The items coming from the character domains are all related to the presence of psychiatric pathologies. The 25 items in question were organized according to the method proposed by the DSM 5, into two dimensions called “passivity” and “Fantasies of Superiority.” Both dimensions describe modes of psychiatric functioning that are clearly maladaptive and unsuitable for managing social relationships and affective and intimate relationships.

These four dimensions have been developed with the aim of identifying the personality structures that make people sensitive or resilient to the psychiatric pathologies of Axis I. Their “external validity” is documented by higher scores in the subgroup of subjects with mental disorders in both clinical samples examined. Only two dimensions of the TCI defined “Harm Avoidance” and “Self-Directedness” present higher values among the subjects with at least one psychiatric disorder but the variance explained by the TR dimensions is greater and this documents an “incremental validity” of the new instrument with respect to the TCI in clinical samples.

As regards forecast validity, the dimensions derived from the temperament “O” and “CE,” were proven to be both sensitive to the presence of the psychiatric pathology of Axis I and capable of predicting its risk. In apparent contradiction with this data, Cloninger’s personality model makes it clear that the temperamental dimensions do not correlate with the pathology while the dimensions of the character in their extreme expressions allow the prediction of the presence of personality disorders and affective and anxious disorders (Cloninger, 2006).

A probable explanation of this difference lies in the fact that the temperamental dimensions of the TR seem to describe strengths (optimistic attitude in front of novelties) or weaknesses (emotional unpleasantness to novelty) in the personality structure, while the two dimensions derived from the character description of the subject’s ways of functioning that are likely to be susceptible to learning-based changes. These four dimensions are interrelated according to a modality that supports the model of insight-based learning according to which the personality structure develops starting from the temperamental dimensions on a dynamic process of circular interaction between temperament and environment. From this point of view, character traits are on the same level as psychiatric disorders that can be interpreted as the product of the cyclic interaction between problematic temperamental dimensions and unfavorable environmental events (Manna, 2012). In this sense, it is also probable that the personality dimensions show greater predictive validity for personality disorders (Cantone et al., 2012; Sperandeo et al., 2016; Maldonato et al., 2017).

Finally, the correlations between the dimensions document a significant reliability of TR-TCI with respect to the validity of content.

The study needs to be continued to further define the psychometric characteristics of the Reduced Test and to evaluate the external validity and prediction of the instrument in relation to personality disorders. It is also necessary to increase the number of people in the sample to confirm the validity of the construct and to specify the accuracy of the predictive capacity of the instrument by evaluating the possible presence of facets associable to the specific Axis I pathologies.

Furthermore, it will be necessary to evaluate the test–retest stability of the reduced trim and confirm the external validity by comparing the TR-TCI with other reagents of assessment of temperament and character and with other Axis I diagnostic tools.

However, even in the current state of the study, it is evident that in clinical populations it is possible to use an agile test with a small pool of items showing a strong association with the presence of Axis I disorders and a significant predictive capacity. Finally, the dichotomous items do not seem to limit the effectiveness of the instrument.

## Ethics Statement

This study was carried out in accordance with the recommendations of “codice etico per la ricerca in psicologia, comitato etico Associazione Italiana di Psicologia” with written informed consent from all subjects. All subjects gave written informed consent in accordance with the Declaration of Helsinki. The protocol was approved by the “comitato etico per la ricerca della SiPGI Postgraduate School in Gestalt Integrated Psychotherapy” D.M.I.U.R.

## Author Contributions

All authors contributed equally to the conception of this work. SD: study design and manuscript writing. EM and RS: data collection and statistics analysis. NMM: study design, critical revisions and supervision.

## Conflict of Interest Statement

The authors declare that the research was conducted in the absence of any commercial or financial relationships that could be construed as a potential conflict of interest.
